# Supplementary material for: Protection against Paraquat-Induced Oxidative Stress by Curcuma longa Extract-Loaded Polymeric Nanoparticles in Zebrafish Embryos
Source: Polymers (Basel). 2022 Sep 9;14(18):3773. doi: 10.3390/polym14183773 (PMC9503139; doi:10.3390/polym14183773)
Supplement: Supplementary file 1 [file polymers-14-03773-s001.zip › polymers-1888392-supplementary.pdf]

SUPPLEMENTARY MATERIAL

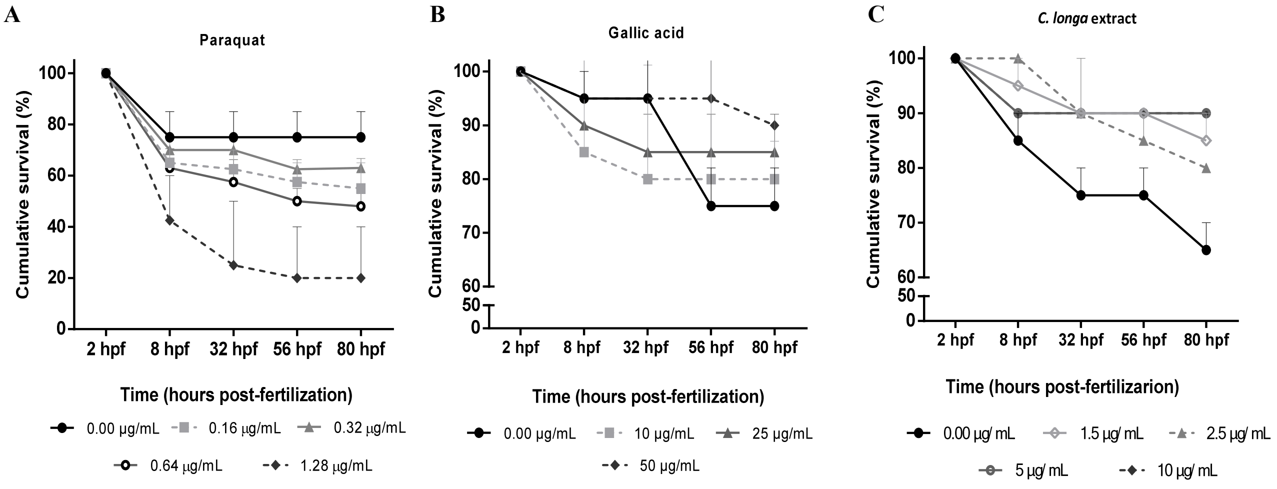

Figure S1. Effects of (**A**) paraquat; (**B**) gallic acid and (**C**) *Curcuma longa* extract on zebrafish embryos at different hours post-fertilization (h_{pf}). Results are expressed as mean ± SD. Chi-square test at 80 h_{pf} for: paraquat - $\chi2$=35.26, *P*<0.05; *C. longa* extract - χ2=23.889, *P*<0.001 and gallic acid - χ2=7.143, *P*=0.067.

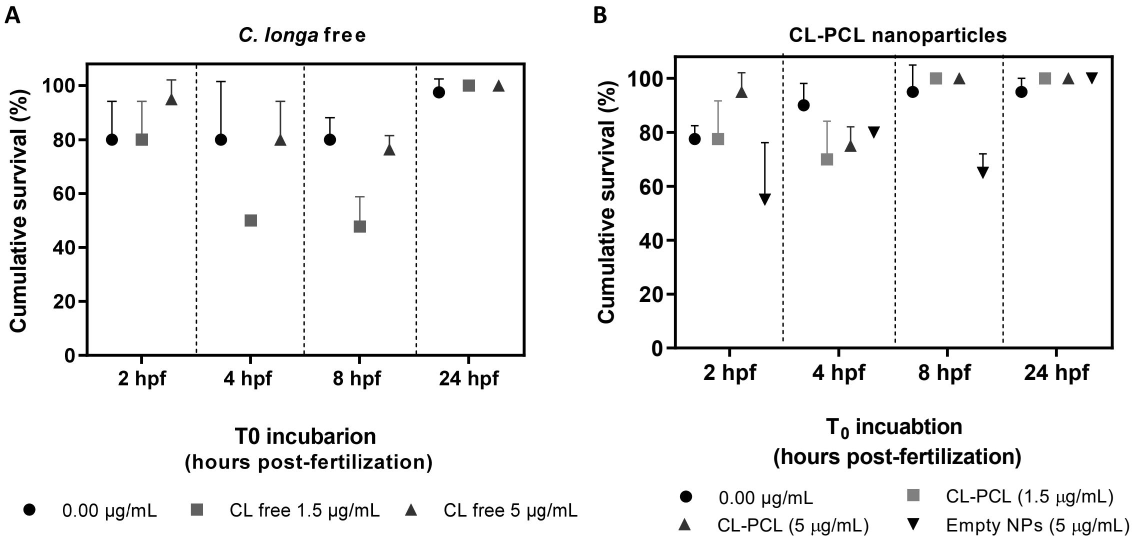

Figure S2. Short exposure effect of *Curcuma longa* extract (CL) free (**A**) and loaded to poly-Ɛ-caprolactone nanoparticles (CL-PCL NPs) (**B**), on survival of zebrafish embryos. 1.5 and 5 µg/mL were the range of concentrations tested. 2, 4, 8 and 24 hours post-fertilization (h_{pf}) were the incubation times. Results are expressed as mean ± SD. Chi square test for CL free: 2 h_{pf}: χ2=42.000, *P*<0.001; 4 h_{pf}: χ2=20.000, *P*<0.001; 8 h_{pf}: χ2=19.494, *P*<0.001; 24 h^{pf}: χ2=5.000, *P*=0.082. Chi square test for CL-PCL NPs: chi square test; 2 h_{pf}: χ2=97.500, *P*<0.001; 4 h_{pf}: χ2=10.294, *P*<0.05; 8 h_{pf}: χ2=85.000, *P*<0.001; 24 h_{pf}: χ2=5.000, *P*=0.082.

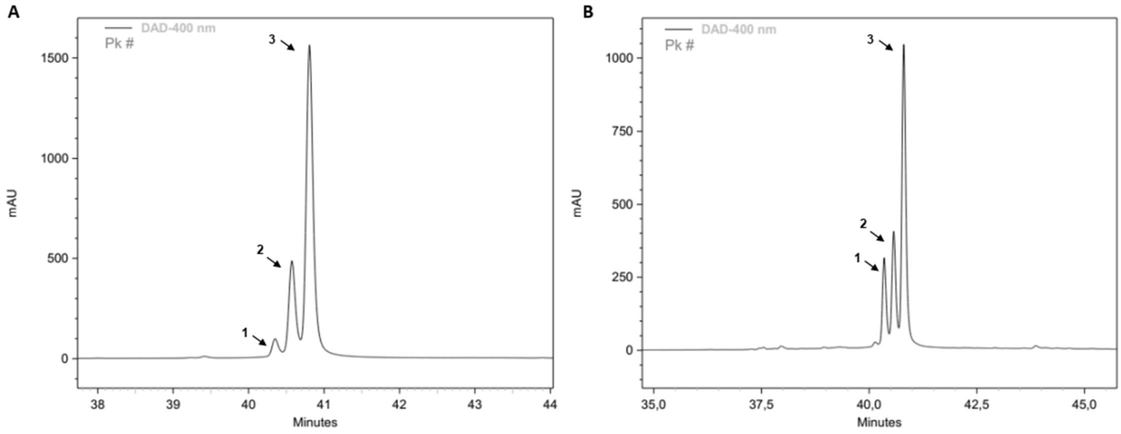

Figure S3: Detailed analysis of high-performance liquid chromatography (HPLC) analysis of commercial standard curcumin (A) and *Curcuma longa* (CL) methanolic extract (B) relating to the period between 38 and 44 min, clearly showing the major compounds present were curcumin (3), demethoxycurcumin (2) and bisdemothoxycurcumin (1).

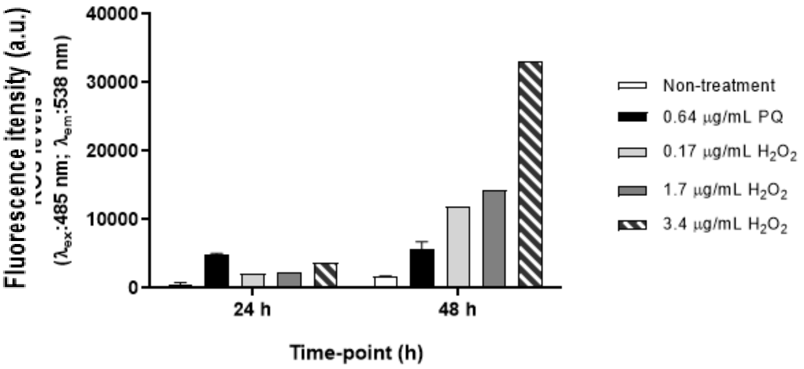

Figure S4. Titration of reactive oxygen species (ROS) induced by paraquat (PQ) and hydrogen peroxide (H_2O_2) exposure on zebrafish embryos. At 2 h_{pf}, the embryos were exposed to 0.64 µg/mL of PQ or 0.17 – 3.4 µg/mL of H_2O_2 for 24 h and, after this incubation time, the media were replaced by freshwater. ROS levels were quantified with DCFH-DA probe (excitation wavelength 485 nm and emission wavelength 538 nm), measured 24 h after incubation (time-point 24 h) and one day after its removal (time-point 48 h).

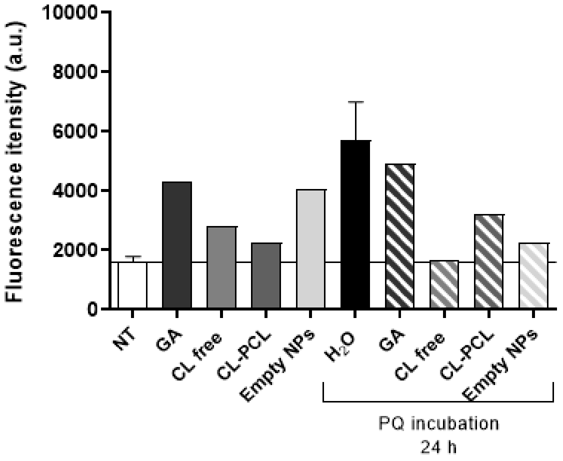

Figure S5. The effects of *Curcuma longa* extract (CL) exposure on paraquat (PQ)-induced ROS generation in zebrafish embryos. Zebrafish embryos were exposed to PQ for 24 h and treated with CL free, CL-poly-Ɛ-caprolactone (CL-PCL) nanoparticles and gallic acid (GA). The concentrations tested were: 0.64 µg/mL PQ; 5 µg/mL CL free, CL-PCL and empty NPs and 25 µg/mL of GA. ROS measurement was performed one day after PQ removal (time-point 48 h).

Table S1. Zebrafish embryotoxicity test – short exposure toxicity assessment. (+) stands for statistically significant effect and (-) for non-statistically significant effect. h_{pf}, hours post-fertilization; CL Free, *Curcuma longa* extract and CL-PCL NPs, *Curcuma longa* extract - poly-Ɛ-caprolactone nanoparticles.

	Independent variables	h_{pf}	CL Free (1.5 µg/mL)	CL-PCL NPs (1.5µg/mL)
**Morphometric parameters**	Yolk volume	2	+	-
		4	+	-
		8	-	-
		24	-	-
	Head-trunk index	2	-	-
		4	-	+
		8	-	-
		24	-	-
	Pupil surface	2	-	-
		4	-	-
		8	-	-
		24	+	-
**Neuro-motor parameters**	Cardiac frequency	2	-	-
		4	-	+
		8	-	+
		24	-	+
	Spontaneous movements	2	+	-
		4	-	-
		8	-	-
		24	-	-

Table S2. Zebrafish embryotoxicity test - post-incubation experiment. (+) stands for statistically significant effect and (-) for non-statistically significant effect.GA [25] - gallic acid 25 µg/mL; GA [50] - gallic acid 50 µg/mL; PQ - paraquat (0.64 µg/mL).

			Post-incubation conditions				
	Independent variables	h_{pf}	GA [25]	GA [50]	PQ ↓ H_2O	PQ ↓ GA [25]	PQ ↓ GA [50]
**Morphometric parameters**	Epibolic arc	8	-	-	-	-	-
	Yolk volume	8	-	-	-	-	-
	Head-trunk index	32	-	-	-	-	+
	Yolk volume	32	-	+	-	-	-
	Pupil surface	32	-	-	-	-	-
	Yolk volume	56	-	+	-	-	-
	Eye surface	56	-	-	-	-	-
**Neuro-motor parameters**	Spontaneous movements	32	+	+	+	+	-
	Cardiac frequency	56	-	-	+	+	+
	Free-swimming	80	-	-	-	-	-
**Cumulative survival**	Survival	80	-	-	+	-	-
